# Genome-wide transcriptome analysis to further understand neutrophil activation and lncRNA transcript profiles in Kawasaki disease

**DOI:** 10.1038/s41598-018-36520-y

**Published:** 2019-01-23

**Authors:** Tai-Ming Ko, Jeng-Sheng Chang, Shih-Ping Chen, Yi-Min Liu, Chia-Jung Chang, Fuu-Jen Tsai, Yi-Ching Lee, Chien-Hsiun Chen, Yuan-Tsong Chen, Jer-Yuarn Wu

**Affiliations:** 1 Institute of Biomedical Sciences, Academia Sinica, Taipei, Taiwan; 2 Department of Biological Science and Technology, National Chiao Tung University, Hsinchu, Taiwan; 3 Graduate Institute of Integrated Medicine, College of Chinese Medicine, China Medical University, Taichung, Taiwan; 4 Department of Pediatrics, China Medical University Hospital, Taichung, Taiwan; 5 College of Medicine, China Medical University, Taichung, Taiwan; 6 School of Chinese Medicine, China Medical University, Taichung, Taiwan; 7 Department of Medical Genetics, China Medical University Hospital, Taichung, Taiwan; 8 Department of Health and Nutrition Biotechnology, Asia University, Taichung, Taiwan; 9 Institute of Cellular and Organismic Biology, Academia Sinica, Taipei, Taiwan; 10 Department of Pediatrics, Duke University Medical Center, Durham, North Carolina USA

## Abstract

Kawasaki disease (KD) is the most common cause of acquired cardiac disease in children in developed countries. However, little is known regarding the role of transcriptomic targets of KD in the disease progression and development of complications, especially coronary artery aneurysms (CAA). The aim of our study was to identify transcripts affected by KD and their potential role in the disease. We enrolled 37 KD patients and collected blood samples along a comprehensive time-course. mRNA profiling demonstrated an abundance of CD177 transcript in acute KD, and in the intravenous immunoglobulin (IVIG)-resistant group compared to in the IVIG-sensitive group. lncRNA profiling identified XLOC_006277 as the most highly expressed molecule. XLOC_006277 expression in patients at acute stage was 3.3-fold higher relative to patients with convalescent KD. Moreover, XLOC_006277 abundance increased significantly in patients with CAA. XLOC_006277 knockdown suppressed MMP-8 and MMP-9 expression, both associated with heart lesions. Our result suggested that the increase of CD177^pos^ neutrophils was associated with KD. Moreover, this study provided global long non-coding RNA transcripts in the blood of patients with KD, IVIG-resistant KD, or CAA. Notably, XLOC_006277 abundance was associated with CAA, which might contribute to further understanding of CAA pathogenesis in KD.

## Introduction

Kawasaki disease (KD) is an acute vasculitis that damages the coronary arteries of infants and young children^1,2^. The first-line treatment for acute KD is high-dose intravenous immunoglobulin (IVIG). This can resolve inflammation and reduce the occurrence of abnormalities in coronary arteries^3^. However, approximately 20% of patients have persistent or recurrent fever after administration of intravenous immunoglobulin and an increased risk of developing a coronary artery aneurysm (CAA). Currently, the causes of KD and the mechanisms that lead IVIG-treatment to increase CAA rates are unknown, although clinical and genetics studies both strongly suggest that an infectious agent triggers KD and genetic predisposition may underlie etiology.

Based on previous studies, it has been proposed that an inflammatory stimulus sets in motion a cascade of events that leads to host immune dysregulation in genetically predisposed individuals. During the acute-phase of KD, multiple immune-related cell populations, including T cells, neutrophils, and macrophages, are all activated. Toxic neutrophils and neutrophil-derived peptides have been observed in acute-phase KD^4^. After IVIG-treatment in IVIG-sensitive patients with KD, immune activation reverts to a normal state. Characterization of the global transcriptome profile during a complete time course of KD, including patients with and without CAA, will aid the identification of novel targets or pathways that are involved in the pathogenesis of KD. It will also contribute to an understanding of the mechanism behind IVIG treatment. One such option is transcriptional profiling of the blood based on a comprehensive, unbiased method.

Previously, it has been shown that the expression levels of certain mRNAs (such as MMP-8, MMP-9, IL-1 beta, and S100A8)^5,6^ and non-coding transcripts (eg., miRNA)^7,8^ can be up-regulated during the acute phase of KD, compared to in healthy controls^9,10^. However, the identity and putative function of transcripts affected by KD remain poorly understood. In addition, the profiles of long (longer than 200 nucleotides in length) non-coding RNAs (lncRNAs) for KD have never been investigated. lncRNAs are involved in many diverse biological processes through the regulation of gene transcription^11^, and have many important roles in human diseases, particularly cancer^12–17^. While lncRNAs have been examined during many clinical studies, none have been examined in acquired inflammatory heart diseases, including KD.

In this study, we performed a comprehensive genome-wide transcriptome expression assessment of three distinct stages during KD. Moreover, we examined the role of KD-associating transcripts with the efficacy of IVIG-treatment and the development of CAA. This will assist future clinical applications and diagnosis.

## Patients and Methods

### Ethical statement

The study was approved by the Institutional Review Board and the Ethics Committee of the Institutional Review Board of China Medical University Hospital Medical Center and Academia Sinica, Taiwan. Written informed consents were obtained from the subjects or their family members in accordance with institutional requirements and Declaration of Helsinki principles. All methods were performed in accordance with the relevant guidelines and regulations.

### Patient groups

Patients were recruited at the China Medical University Hospital Medical Center, Taichung. Thirty-seven KD patients included 30 subjects who were IVIG-sensitive and 7 KD subjects who were IVIG-resistant. KD was diagnosed using previously defined clinical diagnostics, and the definition of IVIG-resistance was based on the criteria used in US^2,18,19^. Coronary artery aneurysms (CAA) were defined as a 50% or greater increase in the diameter of the coronary artery compared to adjacent arterial segments^1,20^. Among the 37 KD cases, 11 cases developed CAA and 26 cases had no observable CAA. We performed two echocardiographic examinations during the acute stage of KD and two months after the onset of symptoms. In pediatric patients less than five years old, CAA was identified when either the left or the right coronary artery exhibited a dilated of diameter ≥3 mm. In older KD patients, CAA was identified when a dilated diameter was shown to be ≥4 mm^18,21^. The baseline demographic summary of CAA group (n = 11) and IVIG-resistant group (n = 7) was shown in the Supplementary Table 1.

### RNA sample collection and preparation

Samples (2 ml) of whole blood were collected in PAXgene Blood RNA collection tubes (Qiagen). These prevent degradation of labile mRNAs by inhibiting RNases at the time of phlebotomy^22^. Total RNA was extracted and purified per the manufacturer’s instructions.

### Microarray analysis

SurePrint G3 Human Gene Expression (Agilent Technologies) was applied in this study. This array could provide comprehensive coverage of genes and completely cover the catalog of lncRNAs from the LNCipedia 2.1 database. For the microarray experiments, 500 ng of total RNA was amplified using a Low Input Quick-Amp Labeling kit (Agilent Technologies) and labeled with either Cy5 or Cy3 (PerkinElmer) during the *in vitro* transcription process. Raw image data were extracted using Feature Extraction 10.5.1.1 software (Agilent Technologies). To normalize data, a LOWESS method was used to subtract any background and scale the average signal intensity for each sample to the global average signal intensity. The log2 Cy5/Cy3 ratio of a specific locus was applied to normalize any differential expression of the two dyes used. The gene expression analysis software GeneSpring GX (Agilent Technologies) was used to perform further normalization. To identify transcripts with differentially expression in the acute stage of KD (acute versus recovery), two-way analysis of variance (ANOVA) was performed. Two factors of ANOVA analysis were the “stage” (“acute” versus “recovery”) and “type” (KD versus non-KD). The transcripts with p < 0.05 were then selected into a pool of acute-KD-related transcripts. We sorted the top 50 mRNA targets and the top 50 lncRNA targets from a pool of acute-KD-related transcripts based on their folds of expression (acute versus recovery).

### Quantitative RT-PCR

Total RNA from patients with KD was isolated and reverse transcribed to cDNA using Superscript III reverse transcriptase (Invitrogen). Quantitative real-time PCR reactions were performed on the ABI Prism 7900HT Sequence Detection system and fluorescent signal intensity was analyzed using Sequence Detector v2.3 software (Applied Biosystems). The relative abundances of target transcripts were normalized to the expression level of a housekeeping gene, GAPDH, or the neutrophil marker CD16, using the ΔΔCt relative quantification method. Two-tailed unpaired student’s t-tests were performed to identify any statistically significant differences in gene expression between regions.

### Separated cell isolation and RNA extraction

Peripheral blood polymorphonuclear leukocytes (PMNs) were isolated from heparinized venous blood using Ficoll-Histopaque (Sigma) gradient centrifugation. Neutrophils (CD16^+^) were isolated sequentially using Dynabeads per manufacturer’s instructions. RNA was extracted from whole blood (PAXgene Blood RNA Kit) or cell populations (The RNAqueous®-Micro Kit) and stored at −80 °C until use.

### Flow cytometry

We performed immunophenotypic analysis using distinct fluorochrome-conjugated monoclonal antibodies that recognized human CD177 (MEM-166 clone, eBioscience), or CD16 (eBioCB16, eBioscience). Cells were examined by multicolor flow cytometry using a FACSCalibur machine (BD Biosciences), and data were analyzed using CellQuest acquisition software. The CD16^+^CD177^+^ neutrophils cells were isolated by FACS sorting on a BD FACSAria system (BD Biosciences).

### Knockdown of XLOC_006277 in PBMCs isolated from patients with acute KD

The sequences of functional siXLOC_006277 using the Accell siRNA delivery system (Dharmacon) were designed in four regions: AGACAAAUUUCAAGAGUGAUU, GGCAAUUUAUGGAGACAAAUU, ACUCAAGGCAGGAUAGUUAUU, and CCAAAGAAGCUGUGGGUACUU respectively. Accell Non-targeting Control Pool (Dharmacon), containing non-targeting siRNAs with no homology to known human, mouse, or rat genes, was used as a control in all siRNA transfection experiments. Isolated PBMCs were transfected with siXLOC_006277 using the Accell siRNA delivery system, per the manufacturer’s protocol. Before transfection, cells were cultured with growth media (RPMI medium supplemented with 10% FBS) for 24 hours. Cells were then spun down and the supernatant replaced with Accell delivery media (4 × 10^6^ cells/ml). A 100 μM siRNA solution was prepared by mixing siXLOC_006277 with delivery media for 90 minutes, then 10 μl was added to 1 ml of cells. To each well of 96-well plate, 100 μl of the delivery mix plus cells was added. Cells were cultured at 37 °C with 5% CO_2_ for 72 hours.

## Results

### Globally affected transcript profiles from multiple stages of Kawasaki disease

**Figure 1:**
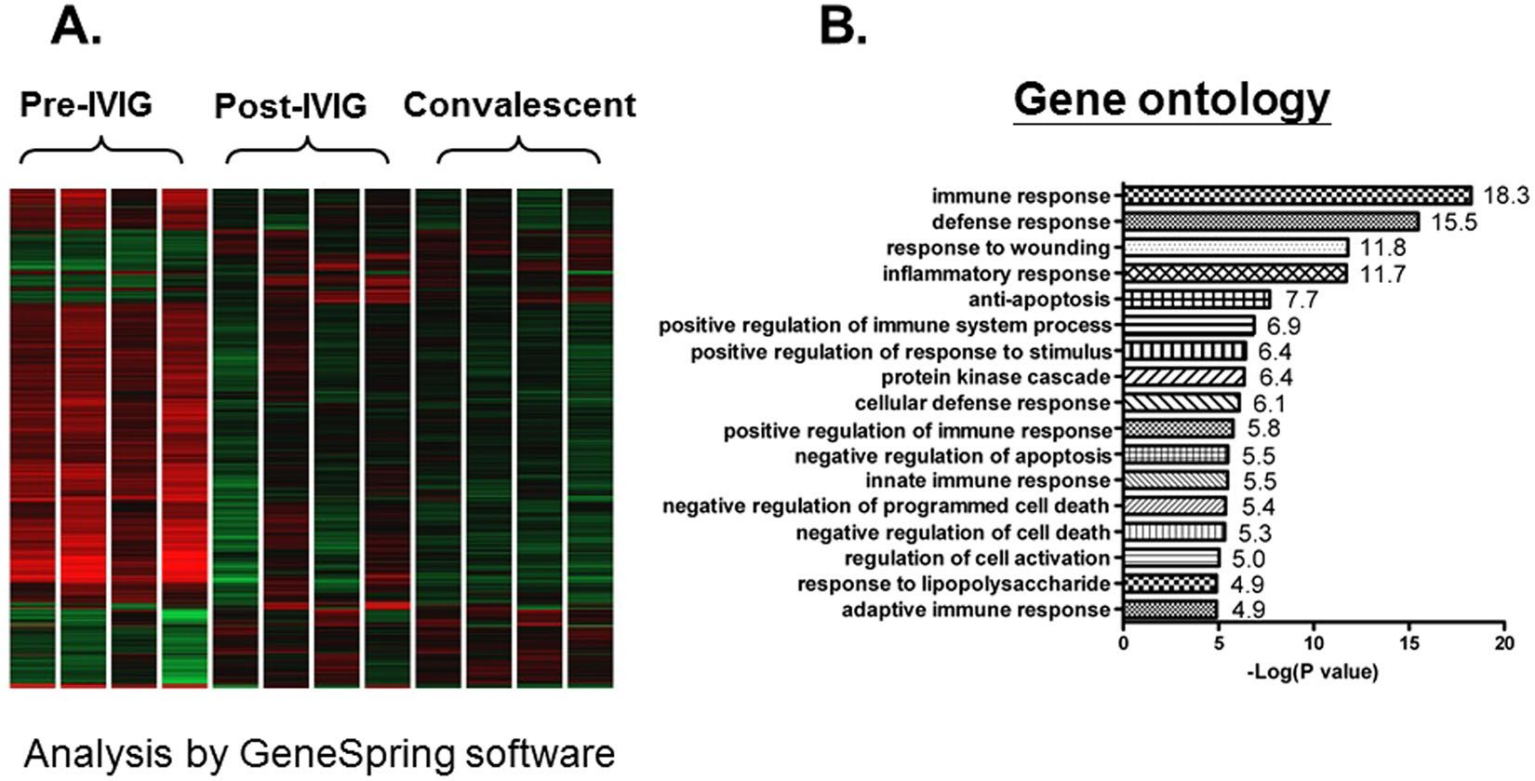
Transcriptome analysis of patients with KD during the acute (Pre-IVIG), subacute (Post-IVIG), and convalescent stages. Graphical representation (**A**) of hierarchical clustering by GeneSpring. Shown are mean changes in the whole transcript levels obtained from whole blood. The 962 transcripts differentially expressed in whole blood of acute KD patients, which was compared with convalescent stage. Blue color regions indicated genes with low expression, and red color regions indicated genes with high expression. Heatmap rows, genes; columns, participants. Gene ontology analysis (**B**) represents potential pathways involved in KD (n = 4).

To identify globally affected expression profiles in the acute stage of KD (Fig. 1A), we performed microarray analysis of total RNA in the acute (before first IVIG treatment), sub-acute (one to seven days after starting IVIG treatment), and the convalescent stages (after two months of IVIG treatment). Gene Ontology analysis (Fig. 1B) revealed that genes involved in the immune response (*−log p* = 18.3), defense response (−*log p* = 15.5), response to wounding (−*log p* = 11.8), and inflammatory response (−*log p* = 11.7) were highly enriched. Using a combination of expression-level and statistical filters, we identified 50 candidate transcripts that were significantly expressed in the acute stage (Supplementary Table 2). Among these transcripts with *p* values lower than 0.001, CD177 had the largest fold change between the acute and recovery stages.

### Top affected mRNA transcript profiles in Kawasaki disease

**Figure 2:**
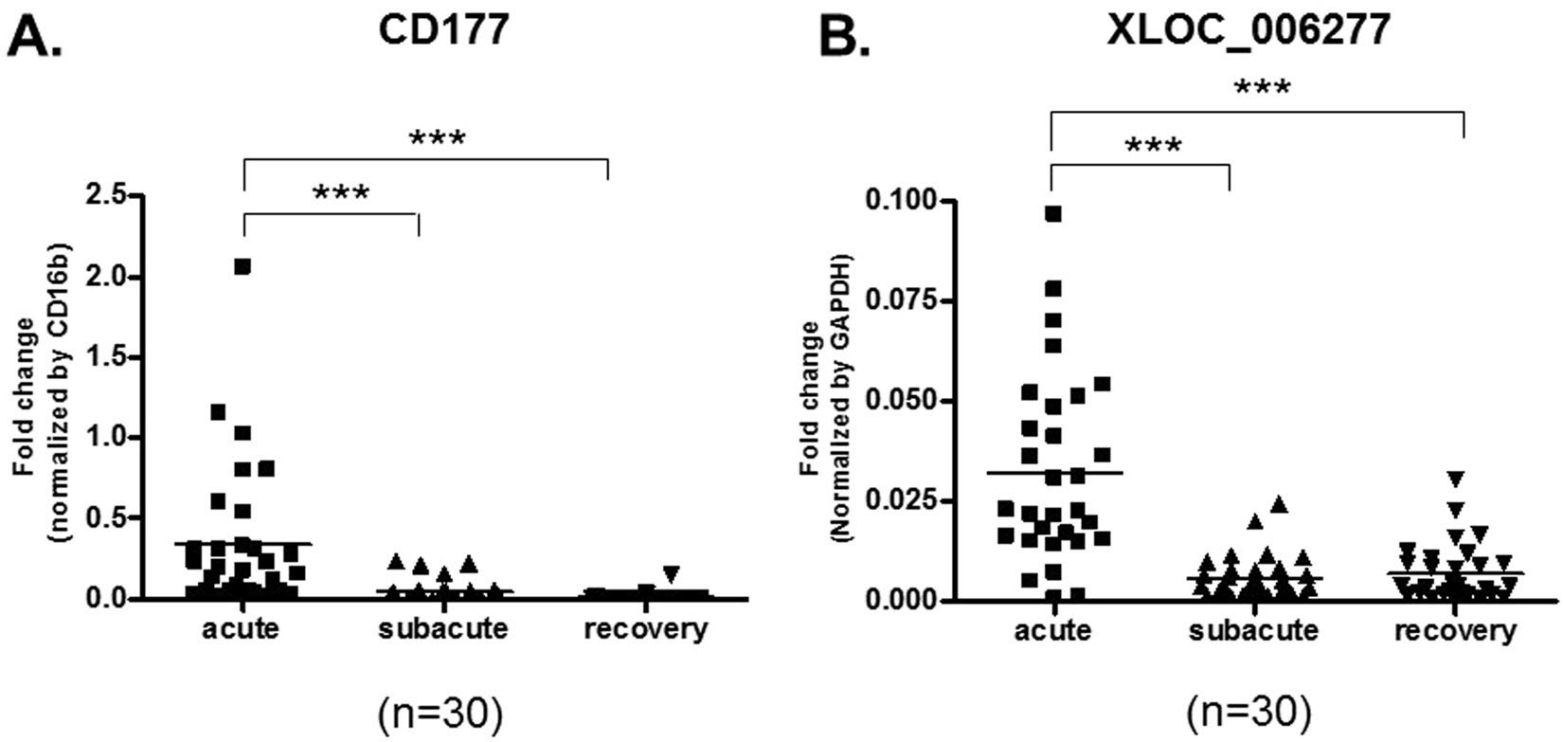
Expression levels of the mRNA transcripts among subjects who responded to IVIG therapy. The expression levels of CD177 (**A**) and XLOC_006277 (**B**) before IVIG (acute), two days after IVIG (subacute), and two months after IVIG therapy (recovery) in KD patients (n = 30) were measured by real-time PCR. Results are presented as the relative units of each transcript compared to CD16b.

**Figure 3:**
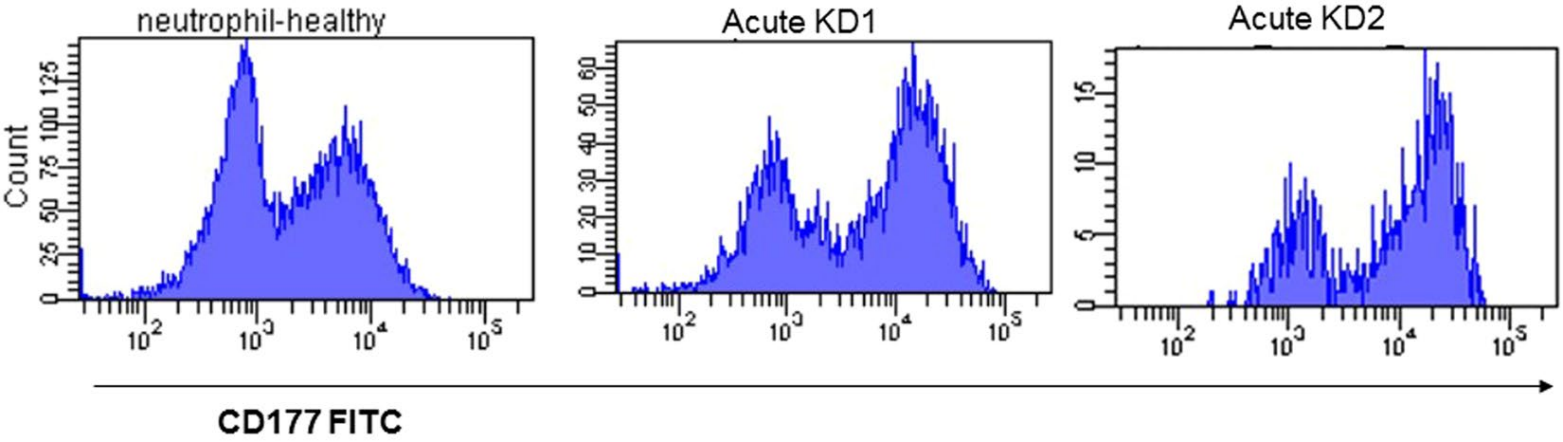
Detection of surface expression levels of CD177 in neutrophils. Membrane expression of CD177 and CD16 on neutrophils was measured in parallel using flow cytometry, and data were shown by 2 to 3 independent tests.

**Figure 4:**
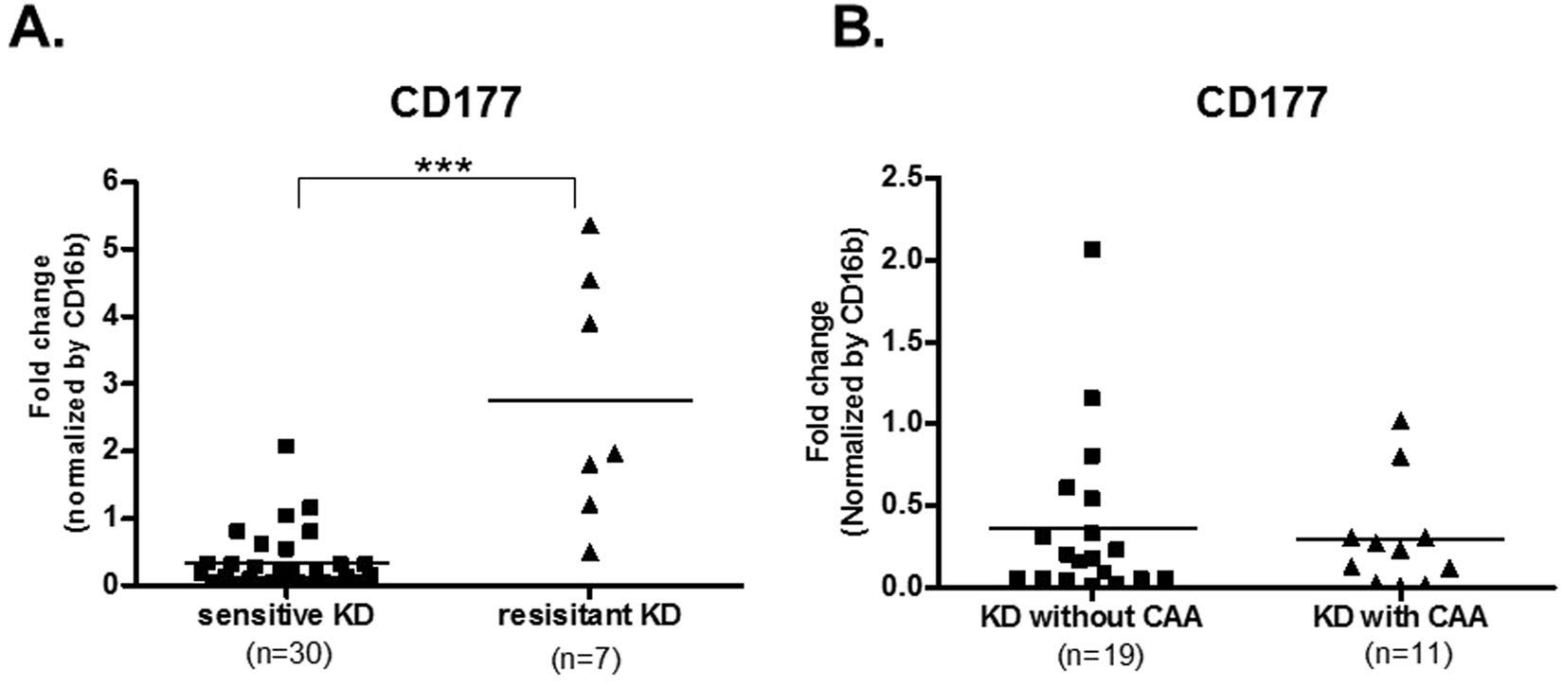
Potential role of CD177 in the efficacy of IVIG and severity of CAA. (**A**) The expression levels of CD177 before IVIG-treatment in patients with KD, who were IVIG-sensitive or IVIG-resistant. (**B**) The expression levels of CD177 before IVIG-treatment in the patients with KD, with or without CAA.

To examine whether the highly expressed transcript, CD177, correlated with the efficacy of IVIG or the development of KD complications (such as CAA), we compared levels of CD177 transcript in blood obtained from patients with KD before IVIG-treatment. CD177 is a surface protein commonly expressed on neutrophils^23^, we therefore used the neutrophil specific marker CD16b as normalizing marker to compensate for an observed increase in the number of neutrophils during the acute stage of KD. This revealed a significant increase in the levels of CD177 transcript and the intensity of surface protein expression on neutrophils in the acute stage of KD (Figs 2A and 3). Comparing the IVIG-sensitive and IVIG-resistant groups, CD177 transcript in the IVIG-resistant group was significantly higher (9-fold) than in the IVIG-sensitive group (Fig. 4A). However, we did not find any difference in the levels of CD177 between the CAA and non-CAA groups (Fig. 4B).

### Top expressed lncRNA transcript profiles in Kawasaki disease

**Figure 5:**
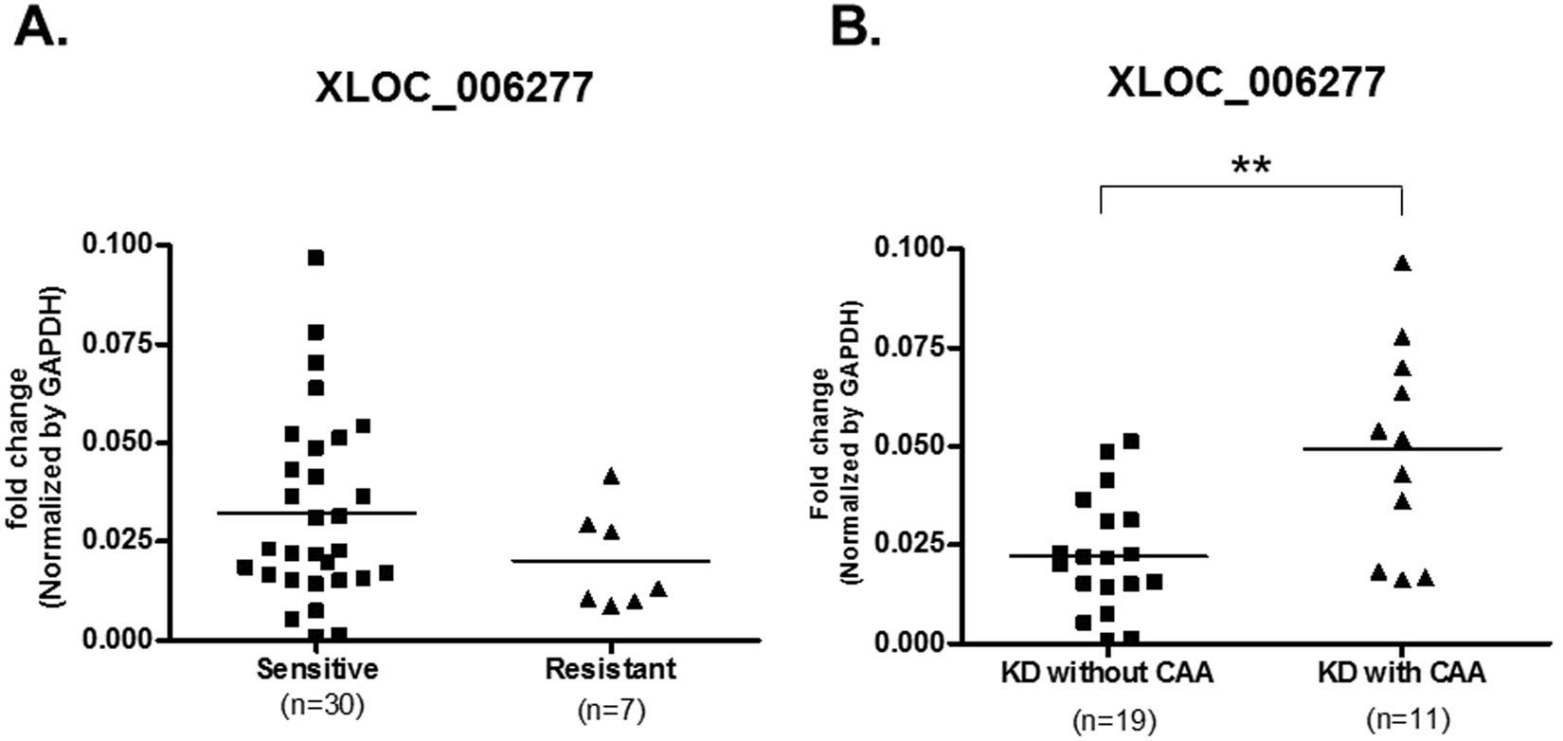
Potential impact of XLOC_006277 in the efficacy of IVIG and severity of CAA. (**A**) The expression levels of XLOC_006277 before IVIG-treatment in patients with KD who were IVIG-sensitive or IVIG-resistant. (**B**) The expression level of XLOC_006277 before IVIG-treatment in patients with KD, with or without CAA.

**Figure 6:**
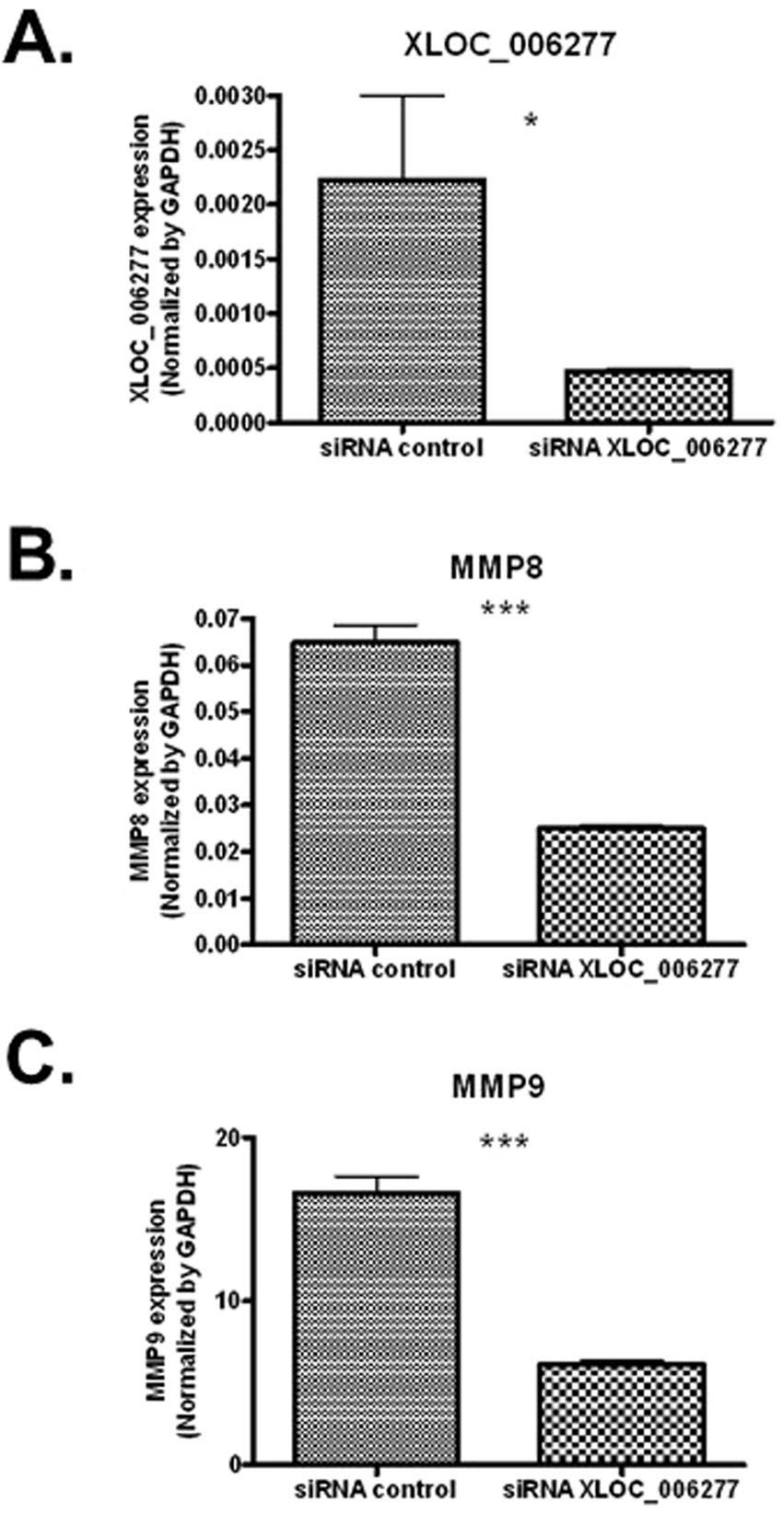
Effects of XLOC_006277 on gene expression in human PBMCs. The effects of siXLOC_006277 against XLOC_006277 were analyzed by quantitative real-time PCR (qRT-PCR). The strongest inhibitory effect was achieved using siXLOC_006277. The expression levels of mRNA for XLOC_006277 (**A**), MMP-8 (**B**) and MMP-9 (**C**) were determined after knockdown of XLOC_006277 by siRNA. Results were normalized to that of GAPDH in each group. Values are the mean ± SEM of triplicate experiments from one patient. *P < 0.05; **P < 0.01; ***P < 0.001 versus negative control (treated with Accell non-targeting siRNA).

To identify lncRNAs that associate with KD, we also performed lncRNA transcript analysis in the acute (before first IVIG treatment), sub-acute (one to seven days after starting IVIG treatment), and convalescent stages (after two months of IVIG treatment). Using a combination of expression-levels, statistical filters, and hierarchical clustering, we identified 50 top candidate lncRNA transcripts that were significantly expressed in the acute stage (Supplementary Table 3). To confirm the accuracy of the microarray data, the top differentially expressed lncRNA transcript, XLOC_006277, was validated by quantitative real-time-PCR. When comparing the CAA and non-CAA groups, XLOC_006277 transcript was significantly higher (2.23-fold) in the former group (Fig. 2B) (p < 0.00001). However, we did not find any difference between the IVIG-resistant and IVIG-sensitive groups (Fig. 5B). To analyze any link between XLOC_006277 and the likelihood of developing KD, we examined the effect of XLOC_006277 knockdown on PBMCs isolated from patients with acute KD. We determined the mRNA expression level of MMP-8 and MMP-9 because they have been found to be associated with CAA. After treatment with the siRNA Accell-siXLOC_006277, XLOC_006277 expression was downregulated 21.24% (*p* = 0.02785) (Fig. 6A). The expression of mRNA for MMP-8 and MMP-9 were also significantly decreased by 2.59-fold (*p* = 0.00046) and 2.70-fold (*p* = 0.00047), respectively (Fig. 6B,C).

## Discussion

Kawasaki disease (KD) is the most common acquired heart disease in children worldwide, including Taiwan^24^. Despite advances in elucidating the genetics involved in KD, a comprehensive understanding of any transcriptomic basis remains unclear. Genome-wide gene expression screening provides new opportunities to understand pathogenic mechanisms, to develop novel diagnostics, prognostic markers, or to reveal new therapeutic avenues. Molecular phenotyping of diseases using high-dimensional gene expression technologies, such as high-density microarrays, can be used to comprehensively survey the global transcript expression profile during a disease. In the present study, we compared global mRNA expression profiles in whole blood from patients with acute KD, patients with IVIG-resistance, and patients with CAA, using both microarray. Similar to previous studies^25^, we confirmed that the up-regulation of *CD177* transcript observed in KD, was also associated with IVIG efficacy. In addition, because CD16 expression is abundant on eosinophils whose numbers are elevated in the peripheral circulation in acute and sub-acute KD, the upregulation of CD177 (normalized by CD16) are expected to be much higher in acute KD after diminishing the contribution of CD16 expression from eosinophils. As it has previously been reported that CD177 is up-regulated on neutrophils after stimulation, such as during severe bacterial infections^26^, it was hypothesized that CD177 may be involved in the processes of neutrophil-mediated host defense^27^. Consistent with our pathway analysis, we also identified bacterial infections highly associated with KD.

Having demonstrated the altered expression of certain mRNAs and miRNAs during KD^25,28–30^, we proceeded to investigate whether the disease also associated with changes in expression of lncRNAs. Despite their widespread expression, the importance of lncRNAs in the regulation of multiple physiological and pathological responses has only recently emerged. Using a transcriptomics-based approach, we identified an alteration in lncRNA expression in the blood from patients with acute KD. In addition, the top differentially expressed lncRNA transcript, XLOC_006277, was significantly up-regulated in acute KD and might be correlated with the UDP-galactosyltransferase activity^31^. More importantly, higher levels of XLOC_006277 transcript were found in patients with KD that later developed CAA. After knockdown of XLOC_006277 with siRNA, KD or CAA-related markers, such as MMP-8 and MMP-9, were down-regulated^32,33^. Therefore, our data support the hypothesis that XLOC_006277 is critical for upstream signals that associate with CAA and is potentially a novel biomarker to predict CAA development.

In summary, our mRNA profiling suggests that neutrophils may be involved in the pathogenesis of KD, and therapies that suppress neutrophil activation and gene expression in peripheral blood may prove effective. Long non-coding RNA profiling revealed that the long non-coding transcript XLOC_006277 was overrepresented in patients with acute KD and decreased after IVIG in responsive patients. Notably, XLOC_006277 abundance was significantly higher in patients with CAA compared to in those without CAA. These findings provide the first long non-coding RNA transcriptomic profiling for KD and offer a novel critical long non-coding RNA target, XLOC_006277, to investigate the pathogenesis of CAA in KD.

## Electronic supplementary material

Supplementary Materials
