# Supplementary material for: Genome-wide transcriptome analysis to further understand neutrophil activation and lncRNA transcript profiles in Kawasaki disease
Source: Sci Rep. 2019 Jan 23;9:328. doi: 10.1038/s41598-018-36520-y (PMC6344526; doi:10.1038/s41598-018-36520-y)
Supplement: Supplementary file 1 — Supplementary Materials [file 41598_2018_36520_MOESM1_ESM.pdf]

Original Article

Genome-wide transcriptome analysis to further understand neutrophil activation and lncRNA transcript profiles in Kawasaki disease

Tai-Ming Ko[1,2,3],* Jeng-Sheng Chang[4,5],* Shih-Ping Chen[1], Yi-Min Liu[1], Chia-Jung Chang[1], Fuu-Jen Tsai[6,7,8], Yi-Ching Lee[9], Chien-Hsiun Chen[1], Yuan-Tsong Chen[1,10], Jer-Yuarn Wu[1,6]

[1] Institute of Biomedical Sciences, Academia Sinica, Taipei, Taiwan

[2] Department of Biological Science and Technology, National Chiao Tung University, Hsinchu, Taiwan

[3] Graduate Institute of Integrated Medicine, College of Chinese Medicine, China Medical University, Taichung, Taiwan

[4] Department of Pediatrics, China Medical University Hospital, Taichung, Taiwan

[5] College of Medicine, China Medical University, Taichung, Taiwan

[6] School of Chinese Medicine, China Medical University, Taichung, Taiwan

[7] Department of Medical Genetics, China Medical University Hospital, Taichung, Taiwan

[8] Department of Health and Nutrition Biotechnology, Asia University, Taichung, Taiwan

[9] Institute of Cellular and Organismic Biology, Academia Sinica, Taipei, Taiwan

[10] Department of Pediatrics, Duke University Medical Center, Durham, North Carolina, USA.

***These authors contributed equally to this work:** Tai-Ming Ko, Jeng-Sheng Chang

Correspondence should be addressed to:

Jer-Yuarn Wu, PhD. Institute of Biomedical Sciences, Academia Sinica, 128, Academia Road, Section 2. Nankang, Taipei 11529, Taiwan; e-mail: jywu@ibms.sinica.edu.tw; Phone: +886-2-27899075

Supplementary Table 1. Baseline demographic summary of CAA group (n=11) and IVIG-resistant group (n=7)

CAA Group

Patient ID	Gender	Age (yr)	CRP	LMCA(mm)	RCA(mm)	CAA	No. of IVIG
U997	M	1	7.5	3.0	1.9	+	1
V095	M	1	13.1	3.0	1.9	+	1
V098	F	1	1.6	3.0	1.4	+	1
I571	M	2	4.3	2.7	3.8	+	1
V255	M	2	7.1	3.4	1.8	+	1
V260	M	4	22.9	2.5	2.6	+	1
V078	F	0	11.2	3.0	2.3	+	1
V080	F	0	10.5	2.0	1.7	+	1
V085	M	2	27.5	3.9	3.9	+	1
V086	M	1	11.6	2.3	2.0	+	1
V089	M	2	6.0	2.5	2.5	+	1

IVIG-resistant Group

Patient ID	Gender	Age (yr)	CRP	LMCA(mm)	RCA(mm)	CAA	No. of IVIG
F135	M	2	5.2	2.9	1.7	-	2
F171	M	1	3.3	2.3	1.9	-	2
F175	M	2	5.3	2.4	2.2	-	2
F181	M	2	25.7	2.0	2.1	-	2
F203	M	2	6.7	2.1	1.8	-	2
F210	M	0	11.2	2.0	2.0	-	2
F344	M	2	25.6	2.7	2.4	-	2

Supplementary Table 2. Top 50 mRNA transcripts in acute KD (*P* <0.05), including fold change

GeneSymbol	Acute vs recovery Fold change	P value	GeneSymbol	Acute vs recovery Fold change	P value
CD177	158.0	8.6E-06	C1QA	8.6	1.7E-06
OLFM4	21.6	1.2E-05	VNN1	8.4	2.8E-07
C19orf59	20.0	1.5E-06	ETV7	8.1	1.3E-05
FCGR1B	19.4	1.4E-07	MGAM	7.8	1.1E-05
SLC26A8	18.2	1.8E-06	FAM20A	7.5	8.4E-08
CEACAM1	14.1	3.0E-07	S100P	7.5	4.4E-04
C1QB	14.0	7.3E-07	UPP1	7.2	5.3E-08
ANXA3	14.0	5.7E-07	CR1	7.2	1.9E-07
S100A12	14.0	9.0E-06	GYG1	7.1	4.9E-08
ANKRD22	13.9	1.6E-07	ARG1	7.0	3.4E-06
GPR84	13.7	5.5E-07	ACSL1	6.5	1.1E-07
FFAR3	13.2	6.8E-07	ADM	6.4	1.7E-06
BATF2	13.0	4.5E-06	APOL6	6.4	5.3E-03
BMX	11.8	8.6E-07	AP3B2	6.3	2.5E-07
KREMEN1	10.9	1.9E-06	OSM	6.3	2.6E-07
CLEC4D	10.4	9.2E-06	KCNJ15	6.2	1.3E-05
CYP1B1	9.9	9.0E-06	OPLAH	6.2	3.0E-07
GALNT14	9.7	3.2E-06	GK	6.1	2.7E-07
BPI	9.4	1.1E-05	TREML4	6.0	6.1E-04
IL1R2	9.3	3.3E-06	MS4A4A	6.0	3.6E-05
SERPING1	9.1	4.4E-06	NLRC4	5.8	3.4E-06
AIM2	9.0	4.3E-06	KCNJ2	5.7	3.1E-07
CACNA1E	8.9	1.9E-06	FOLR3	5.7	1.1E-05
ANKRD34B	8.8	3.4E-04	DEFA3	5.7	2.1E-06
LTF	8.6	2.9E-05	ALPL	5.7	6.4E-06

Supplementary Table 3. Top 50 lncRNA transcripts in acute KD (*P* <0.05), including fold change

GeneSymbol	Acute vs recovery Fold change	P value	GeneSymbol	Acute vs recovery Fold change	P value
XLOC_006277	10.9	9.32E-05	LOC100128276	2.8	5.68E-06
CYP1B1-AS1	9.5	1.59E-05	LINC00937	2.8	7.23E-03
SMA4	9.2	3.30E-08	LOC729737	2.8	9.53E-05
ST3GAL4-AS1	7.6	2.88E-07	LINC00999	2.8	1.17E-04
LOC441081	7.0	2.38E-05	LINC01061	2.6	7.93E-05
XLOC_014512	6.9	1.99E-06	BASP1P1	2.6	1.63E-07
LOC731424	6.4	1.17E-05	LOC100288102	2.6	6.56E-05
LOC285696	6.2	1.37E-10	LOC100507191	2.5	7.96E-05
LOC101927686	6.0	6.54E-07	XLOC_006416	2.5	4.09E-05
LOC284751	5.8	2.64E-06	NEAT1	2.4	2.30E-04
LILRA6	5.0	5.20E-06	XLOC_010825	2.4	1.23E-05
FLJ27255	4.9	6.63E-09	DKFZP434F142	2.4	1.38E-04
XLOC_004644	4.7	6.65E-06	XLOC_010782	2.4	1.76E-03
GK3P	4.2	2.04E-08	LOC399715	2.4	4.86E-05
LINC01127	4.1	1.23E-05	GBAP1	2.3	8.42E-05
NRADDP	4.1	1.37E-06	LOC100133331	2.2	4.73E-05
LINC00266-1	4.0	2.70E-06	WWTR1-AS1	2.2	1.75E-04
IL10RB-AS1	3.9	2.94E-06	XLOC_001496	2.1	3.00E-04
LINC01093	3.8	1.51E-03	LINC00265	2.1	7.44E-05
LINC01000	3.4	1.44E-04	LOC729218	2.1	5.68E-05
LOC101928143	3.3	6.92E-05	LINC01002	2.0	6.79E-05
CCDC147-AS1	3.1	3.55E-05	LOC643733	1.9	8.94E-07
XLOC_002473	3.1	8.78E-07	XLOC_001266	1.9	7.16E-08
XLOC_002701	3.0	8.70E-07	FLJ21408	1.9	8.43E-04
MRVI1-AS1	2.8	1.34E-06	NUMB	1.9	7.69E-04
